# Dengue Virus Infection-Enhancing Activity in Serum Samples with Neutralizing Activity as Determined by Using FcγR-Expressing Cells

**DOI:** 10.1371/journal.pntd.0001536

**Published:** 2012-02-28

**Authors:** Meng Ling Moi, Chang-Kweng Lim, Kaw Bing Chua, Tomohiko Takasaki, Ichiro Kurane

**Affiliations:** 1 Department of Virology 1, National Institute of Infectious Diseases, Tokyo, Japan; 2 National Public Health Laboratory, Ministry of Health, Sungai Buloh, Selangor, Malaysia; University of Rhode Island, United States of America

## Abstract

**Background:**

Progress in dengue vaccine development has been hampered by limited understanding of protective immunity against dengue virus infection. Conventional neutralizing antibody titration assays that use FcγR-negative cells do not consider possible infection-enhancement activity. We reasoned that as FcγR-expressing cells are the major target cells of dengue virus, neutralizing antibody titration assays using FcγR-expressing cells that determine the sum of neutralizing and infection-enhancing activity, may better reflect the biological properties of antibodies *in vivo*.

**Methods and Findings:**

We evaluated serum samples from 80 residents of a dengue endemic country, Malaysia, for neutralizing activity, and infection-enhancing activity at 1∶10 serum dilution by using FcγR-negative BHK cells and FcγR-expressing BHK cells. The serum samples consisted of a panel of patients with acute DENV infection (31%, 25/80) and a panel of donors without acute DENV infection (69%, 55/80). A high proportion of the tested serum samples (75%, 60/80) demonstrated DENV neutralizing activity (PRNT_50_≥10) and infection-enhancing activity. Eleven of 18 serum samples from patients with acute secondary DENV infection demonstrated neutralizing activity to the infecting serotype determined by using FcγR-negative BHK cells (PRNT_50_≥10), but not when determined by using FcγR-expressing cells.

**Conclusion:**

Human serum samples with low neutralizing activity determined by using FcγR-negative cells showed DENV infection-enhancing activity using FcγR-expressing cells, whereas those with high neutralizing activity determined by using FcγR-negative cells demonstrate low or no infection-enhancing activity using FcγR-expressing cells. The results suggest an inverse relationship between neutralizing antibody titer and infection-enhancing activity, and that neutralizing activity determined by using FcγR-expressing cells, and not the activity determined by using FcγR-negative cells, may better reflect protection to DENV infection *in vivo*.

## Author Summary

Dengue has become a major international public health concern in recent decades. There are four dengue virus serotypes. Recovery from infection with one serotype confers life-long protection to the homologous serotype but only partial protection to subsequent infection with other serotypes. Secondary infection with a serotype different from that in primary infection increases the risk of development of severe complications. Antibodies may play two competing roles during infection: virus neutralization that leads to protection and recovery, or infection-enhancement that may cause severe complications. Progress in vaccine development has been hampered by limited understanding on protective immunity against dengue virus infection. We report the neutralization activity and infection-enhancement activity in individuals with dengue in Malaysia. We show that infection-enhancement activity is present when neutralizing activity is absent or low, and cross-reactive neutralizing activity may be hampered by infection-enhancing activity. Conventional assays for titration of neutralizing antibody do not consider infection-enhancement activity. We used an alternative assay that determines the sum of neutralizing and infection-enhancement activity in sera from dengue patients. In addition to providing insights into antibody responses during infection, the alternative assay provides a new platform for the study of immune responses to vaccine.

## Introduction

Dengue fever (DF) and dengue hemorrhagic fever (DHF) is caused by infection with dengue virus (DENV), a flavivirus, which consists of four serotypes (DENV-1, DENV-2, DENV-3 and DENV-4). DENV affects up to 100 million people annually living in the tropics and sub-tropical areas. Clinical manifestations of DENV infection ranges from asymptomatic and relatively mild dengue fever (DF), to severe, life-threatening illness, dengue hemorrhagic fever (DHF) and dengue shock syndrome (DSS) [1], [2]. In endemic regions, the risk for developing severe infection was speculated to be higher as compared to non-endemic regions due to the higher possibility of secondary exposure to heterologous DENV serotypes [3], [4]. The number of dengue patients has increased in Malaysia over the past 10 years with 7,103 cases and 45 deaths in 2000, to 41,486 cases and 88 deaths in 2009, to, 46,171 cases and 134 deaths in 2010 [5], [6]. All four DENV serotypes co-circulate in Malaysia [7], [8]. High prevalence of severe dengue virus infections and dengue-related deaths in recent years is speculated to be associated to rapid urbanization and global travel, leading to the spread of dengue virus, and thus to higher prevalence of infected individuals [9]–[11].

Primary infection with one DENV serotype does not confer protection to infection with a heterologous serotype [12], [13]. Epidemiological studies have demonstrated that DHF occurs at a higher rate in secondary infection than in primary infection [14]–[17]. DENV sub-neutralizing, infection-enhancing antibodies induced during primary infection is speculated to play a central role in the pathogenesis of DHF [18]–[21]. During secondary infection, sub-neutralizing antibodies form infectious immune-complexes with DENV, resulting in higher levels of viral progeny in FcγR-expressing cells, a phenomenon known as antibody-dependent enhancement (ADE) [22], [23]. It has been speculated that ADE may play a role not only in causing DHF but in worsening a spectrum of DENV illness [24].

We previously demonstrated that higher neutralizing antibody titers were detected using FcγR-negative BHK cells as compared to FcγR-expressing BHK cells [25]. In the present study, we examined DENV infection-enhancing activity in serum samples with varying levels of DENV neutralizing activity using FcγR-expressing BHK cells.

## Materials and Methods

### Serum samples

Eighty serum samples obtained from 80 residents in Perak, Malaysia were used in the study. Perak is located in north-western region of Peninsular Malaysia, and is endemic for dengue, and other flavivirus infections [26]–[28]. Incidence of DENV infection in Perak was 2,288 in 2010, 2,734 in 2009, and, 4,119 in 2008 [6]. The serum samples were collected in 2008 and were provided by National Public Health Laboratory, Malaysia. Characteristics of the patient population sampled are summarized in Table 1.

**Table 1:** Characteristics of the study populations.

Patient Characteristics^a^	Number (n)
Number of patients (samples collected)	80 (80)
Category of infection	
Primary acute DENV infection^b^	
DENV-1	5
DENV-3	2
Secondary acute DENV infection^c^	
DENV-1	7
DENV-3	11
Others^d^	55

a History of previous flavivirus infection of each patient was not determined.

b DENV infection was confirmed as described in Materials and Methods. Primary DENV infection was defined as serum samples that were negative for neutralizing to all of the four DENV serotypes at serum dilution of 1∶10.

c DENV infection was confirmed as described in Materials and Methods. Secondary DENV infection was defined as serum samples that were positive for neutralizing to any of the four DENV serotypes at serum dilution of 1∶10.

d A total of 55 serum samples from 55 non acute dengue patients were used. Serum samples were derived from the following categories: DENV genome and NS1 antigen negative (n = 34), and chikungunya virus (CHIKV) genome positive (n = 21).

### Ethics statement

The study protocol was approved by the ethics committee of the National Institute Infectious Diseases, Japan (Reference no. 210). Patients were de-identified and study data was analyzed anonymously.

### Dengue diagnostics

Patient serum samples used in the present study were examined for the presence of dengue viral RNA by reverse-transcriptase polymerase chain reaction (RT-PCR), and NS1 antigen by enzyme-linked immunosorbant assay (ELISA). Viral RNA was extracted using High Pure RNA extraction kit (Roche Diagnostics, Germany) and DENV serotypes were determined by serotype-specific reverse transcriptase polymerase chain reaction (RT-PCR) [29]. For serological tests, serum samples were heat-inactivated at 56°C for 30 minutes before use. Detection of the NS1 antigen was performed using Platelia Dengue NS1 Antigen (Bio-Rad Laboratories, France) according to manufacturers' instructions. After reaction was terminated, optical density readings (OD) were obtained with a spectrophotometer at wavelengths of 450 nm/620 nm and the index of each sample was calculated with the following formula: OD of samples/OD of calibrators. The index value of each sample was interpreted according to manufacturer's instructions; index values of <0.9, 0.9–1.1, and, >1.1 were considered negative, equivocal, and positive, respectively [30].

### Viruses and cell lines

Dengue virus type 1 (DENV-1), 01-44-1HuNIID strain (GenBank accession no. AB111070), dengue virus type 2 (DENV-2) D2/Hu/OPD030NIID/2005 strain (GenBank accession no. AB219135), dengue virus type 3 (DENV-3) CH53489 strain (GenBank accession no. DQ863638), and, dengue virus type 4 (DENV-4) TVP360 strain were used. BHK cells, a hamster kidney cell line (Japan Health Science Research Resources Bank, Japan) and FcγR-expressing BHK cells were used. Cells were cultured in Eagle's Minimum Essential Medium (EMEM) (Sigma, USA), supplemented with heat inactivated 10% fetal calf serum (FCS, Sigma) at 37°C in 5% CO_2_. FcγR-expressing BHK cells were cultured in EMEM (Sigma), supplemented with heat inactivated 10% FCS (Sigma) and 0.5 mg/ml neomycin (G418, PAA Laboratories GmbH, Austria) at 37°C in 5% CO_2_ [25].

### Plaque reduction neutralization test (PRNT)

Heat-inactivated human serum samples were serially diluted 2-folds from 1∶5 to 1∶1250 with EMEM/2% FCS. As the amount of serum samples was limited, two replicates were tested for each of the serum samples to four dengue serotype. Virus-antibody mixture was prepared by mixing 25 µl of DENV-1, DENV-2, DENV-3, or, DENV-4 at titers of 2000 PFU/ml with 25 µl of serum samples serially diluted 2 folds from 1∶5 to 1∶1250. For infection with DENV alone, the mixture was prepared by mixing 25 µl of DENV-1, DENV-2, DENV-3, or, DENV-4 strains at titers of 2000 PFU/ml with 25 µl of EMEM supplemented with 10% FCS. After incubation at 37°C for 60 minutes, 50 µl of virus-antibody mixture was inoculated on FcγR-negative BHK and FcγR-expressing BHK monolayers in 12-well plates. The plates were then incubated for 60 minutes at 37°C in 5% CO_2_. After virus absorption, the cells were washed once with 1 ml of EMEM (Sigma) and overlaid with 1 ml EMEM (Nissui Pharmaceutical, Japan) containing 2% FCS (Sigma) and 1% methylcellulose (Wako Pure Chemical Industries, Japan). The plates were incubated at 37°C in 5% CO_2_ for 5–7 days, when plaque formation could be confirmed by naked eye. Cells were then fixed with 10% formalin (Wako Pure Chemical Industries) and stained with methylene blue (Wako Pure Chemical Industries). Number of plaques was counted with naked eye. Plaque-reduction neutralizing test (PRNT_50_) end points are expressed as the last serum dilution showing a 50% or greater reduction in plaque counts as compared to the number of plaques determined from wells of cells infected in the absence of antibodies [31].

### Antibody-dependent enhancement (ADE) assay

For enhancement assay against 4 DENV serotypes, 25 µl serum samples diluted at 1∶5 with EMEM/2% FCS were used. Virus-antibody mixture was prepared by mixing 25 µl of DENV-1, DENV-2, DENV-3, or, DENV-4 strains at titers of 1000–2000 PFU/ml with 25 µl of 1∶5 diluted serum samples and incubated at 37°C for 60 minutes. For negative controls, virus mixture was prepared by mixing 25 µl of DENV-1, DENV-2, DENV-3, or, DENV-4 strains at titers of 1000–2000 PFU/ml with 25 µl of 2% FCS/EMEM and incubated at 37°C for 60 minutes. Fifty microliters of the virus-antibody mixture was then applied to FcγR-expressing BHK monolayers in 12-well plates. The plates were then incubated for 60 minutes at 37°C in 5% CO_2_. After virus absorption, the cells were washed with 1 ml of EMEM and overlaid with 1 ml EMEM containing 2% FCS and 1% methylcellulose. The plates were incubated at 37°C in 5% CO_2_ for 5 days. Cells were then fixed with 10% formalin and stained with methylene blue. Plaques were counted with naked eye. Fold enhancement values were determined using the following ratio: (mean plaque count at 1∶10 serum dilution)/(mean plaque count in the absence of human serum samples, negative control). The sum of the mean of the negative control plus two times of the standard deviation (SD) value obtained from 4 wells of negative control was used as cut-off to differentiate enhancing and non-enhancing activity [32], [33]. Enhancing activity was defined as positive when values are greater than the mean plaque count in the absence of human serum samples plus a greater than 2 times SD.

## Results

### Neutralizing and infection-enhancing activities of serum samples collected in Perak, Malaysia

Eighty serum samples were tested for the presence of neutralizing antibody to each of the four DENV serotypes by using BHK cells (Table 1). Neutralizing antibody (PRNT_50_≥10) was detected in 69% (55/80) to DENV-1, 60% (48/80) to DENV-2, 56% (45/80) to DENV-3 and, 20% (16/80) to DENV-4. Of the 80 samples, 12 samples and 13 samples were obtained from patients with acute DENV-1 and DENV-3 infections, respectively (Table 1). Neutralizing activity to DENV-1 was detected in 58% (7/12) of the DENV-1 infected patients and that to DENV-3 was detected in 62% (8/13) of the DENV-3 infected patients. In contrast, enhancing-activity to DENV-1 was detected in 33% (4/12) of the DENV-1 infected patients and that to DENV-3 was detected in 61% (8/13) of the DENV-3 patients (Table 2). Five DENV-1 infected patients and two DENV-3 infected patients demonstrated neither neutralizing nor infection-enhancing activity to any of the four DENV serotypes (Table S4). Four (58%) of the 7 serum samples from acute secondary DENV-1 infected patients demonstrated infection-enhancing activities to DENV-1 serotype and similarly, 8 (73%) of 11 serum samples from acute secondary DENV-3 infected patients enhanced DENV-3 infection. The results indicate that serum samples from dengue patients and non-dengue patients in dengue endemic region, Malaysia, possess neutralizing and infection-enhancing activities (Table S1, Table S2). The history of past dengue infections of each of the patients was however, not known. Because Malaysia is endemic for dengue infection, and some of the serum samples exhibited high levels of neutralizing antibody titers to DENV, it is highly likely that the patients with high neutralizing antibody activity has been previously exposed to DENV infection.

**Table 2:** Neutralizing and infection-enhancement activities against each of the 4 serotypes of dengue virus.

Activity	% Prevalence of neutralizing or enhancing activity to DENV serotypes(number positive/total number)			
	DENV-1	DENV-2	DENV-3	DENV-4
Neutralizing antibody (PRNT_50_ ≥1∶10)	69 (55/80)	60 (48/80)	56 (45/80)	20 (16/80)
DENV-1 patient^a^	58 (7/12)	67 (8/12)	17 (2/12)	8 (1/12)
DENV-3 patient^a^	69 (9/13)	69 (9/13)	62 (8/13)	15 (2/13)
Others^b^	71 (39/55)	56 (31/55)	64 (35/55)	69 (38/55)
Enhancing activity^c^	26 (21/80)	26 (21/80)	30 (24/80)	73 (58/80)
DENV-1 patient^a^	33 (4/12)	25 (3/12)	42 (5/12)	75 (9/12)
DENV-3 patient^a^	38 (5/13)	8 (1/13)	62 (8/13)	85 (11/13)
Others^b^	22 (12/55)	31 (17/55)	25 (14/55)	69 (38/55)

a Serum samples obtained from DENV patients. Infecting DENV serotype was determined by RT-PCR.

b Others consist of a total of 55 serum samples from 55 non acute dengue patients. Serum samples were derived from the following categories: DENV genome and NS1 antigen negative (n = 34), and chikungunya virus (CHIKV) genome positive (n = 21).

c Positive infection-enhancement activity was defined as fold-enhancement values greater than cut-off plus a 2 times SD (or cut-off+2SD) in the mean plaque count in the presence of human serum samples.

### Difference in neutralizing antibody titers to 4 DENV serotypes determined by using FcγR-negative BHK cells and FcγR-expressing BHK cells

The 18 samples derived from acute secondary dengue patients which demonstrated both neutralizing and infection-enhancing activities were examined for neutralizing antibody titers to each of the 4 DENV serotypes. The serum samples were from 7 acute secondary DENV-1 patients and 11 acute secondary DENV-3 patients (Table 1). Using FcγR-negative BHK cells, 7 serum samples possessed neutralizing antibody titers only to DENV-2 and 11 samples showed neutralizing antibody to more than one serotype (Table 3, Table S3). All the serum samples except one (17 of 18) demonstrated neutralizing antibody titers to DENV-2 at titers of 1∶10 to 1∶1280. Using FcγR-positive BHK cells, 13 serum samples obtained from patients with DENV-1 or DENV-3 infection possessed neutralizing antibody titers only to DENV-2 (1∶10 to 1∶160), and 2 samples possessed neutralizing antibody titers only to DENV-1 (1∶10 to 1∶20). Some of the non acute dengue patients (serum sample no. 23, 28, 30, 38, 73, 74, 77, 78, 79, Table 4) exhibited heterotypic neutralizing activity using BHK cells but monotypic neutralizing activity using FcγR-expressing cells, as those observed in patients with acute secondary dengue infection (Table 3).

**Table 3:** Level of neutralizing activity in serum samples obtained from patients with secondary acute dengue infection and from selected non acute dengue patients against each of the 4 DENV serotypes.

Patient^a^	Patientno	Neutralizing antibody titer to DENV (PRNT_50_)							
		BHK cells				FcγR-expressing BHK cells			
		DENV-1	DENV-2	DENV-3	DENV-4	DENV-1	DENV-2	DENV-3	DENV-4
**Infecting serotype: DENV-1** b	**46**	80	40	20	20	<10	10	<10	<10
	**47**	<10	20	<10	<10	<10	<10	<10	<10
	**48**	<10	10	<10	<10	<10	<10	<10	<10
	**49**	10	1280	<10	<10	<10	160	<10	<10
	**56**	10	10	<10	<10	<10	<10	<10	<10
	**57**	<10	40	<10	<10	<10	40	<10	<10
	**58**	<10	320	<10	<10	<10	10	<10	<10
**Infecting serotype: DENV-3** b	39	80	<10	10	<10	20	<10	<10	<10
	**40**	20	160	20	<10	<10	40	<10	<10
	**41**	<10	80	<10	<10	<10	20	<10	<10
	**42**	20	640	40	40	<10	80	<10	<10
	**43**	80	40	20	<10	10	<10	<10	<10
	**44**	10	320	10	<10	<10	80	<10	<10
	**45**	40	320	40	<10	<10	80	<10	<10
	**52**	20	20	10	<10	<10	10	<10	<10
	**53**	<10	160	<10	<10	<10	40	<10	<10
	**54**	80	1280	40	10	<10	80	<10	<10
**Non acute DENV** c	**15**	20	<10	10	<10	<10	<10	<10	<10
	**23**	20	160	160	<10	<10	80	<10	<10
	**28**	40	<10	40	10	20	<10	<10	<10
	**30**	40	40	20	10	<10	20	<10	<10
	**38**	<10	320	<10	<10	<10	80	<10	<10
	**73**	80	<10	20	<10	20	<10	<10	<10
	**74**	40	<10	80	10	20	<10	<10	<10
	**77**	80	10	10	<10	40	<10	<10	<10
	**78**	10	80	10	<10	<10	40	<10	<10
	**79**	10	10	160	<10	<10	<10	40	<10

a Infecting serotype indicates the DENV serotype detected in the serum sample as determined by RT-PCR.

b Neutralizing titers (PRNT_50_) to each of the 4 DENV serotypes for five DENV-1 patients and for two DENV-3 patients were less than 10 (PRNT_50_<10) by using FcγR-negative BHK cells and FcγR-expressing BHK cells and the results were not included in Table 3. Neutralizing antibody titer to 4 DENV serotypes were determined by a conventional PRNT method using FcγR-negative BHK cells and FcγR-expressing BHK cells as indicated in Materials and Methods.

c Neutralizing antibody titers were determined for samples from selected non acute dengue patients.

**Table 4:** Dengue virus enhancement activities in serum samples obtained from patients with secondary dengue infection and from selected non acute dengue patients.

Patient^a^	PatientNo.	Fold enhancement^b^			
		DENV-1	DENV-2	DENV-3	DENV-4
**Infecting serotype: DENV-1**	**46**	0.7	0.1	0.9	5.6
	**47**	5.6 c	2.1	5.1	1.1
	**48**	4.7	2.0	5.3	4.4
	**49**	1.2	<0.1	1.0	2.0
	**56**	2.8	1.6	4.2	6.9
	**57**	1.2	<0.1	1.5	4.7
	**58**	5.3	<0.1	4.9	6.0
**Infecting serotype: DENV-3**	**39**	<0.1	2.3	1.5	6.1
	**40**	1.9	<0.1	1.1	4.6
	**41**	5.5	<0.1	5.7	6.5
	**42**	0.6	<0.1	1.7	1.4
	**43**	0.5	0.9	1.9	5.3
	**44**	1.5	<0.1	4.3	7.3
	**45**	0.7	<0.1	0.8	5.9
	**52**	1.5	<0.1	1.0	0.9
	**53**	5.2	0.2	1.4	4.7
	**54**	0.8	<0.1	2.0	5.0
	**55**	5.2	0.2	6.7	6.9
**Non acute DENV** d	**15**	1.0	4.1	4.3	5.4
	**23**	2.5	<0.1	1.3	4.4
	**28**	0.2	2.4	4.1	5.5
	**30**	3.2	0.1	2.5	6.1
	**38**	5.1	<0.1	6.3	5.6
	**73**	<0.1	1.5	4.3	7.3
	**74**	<0.1	1.2	1.1	2.5
	**77**	<0.1	1.9	1.3	6.7
	**78**	1.7	<0.1	1.3	4.7
	**79**	1.8	1.4	<0.1	6.6

a Infecting serotype indicates the DENV serotype detected in the serum sample as determined by RT-PCR.

b Fold enhancement values are enhancement ratio calculated by the fornula: mean plaque count at 1∶10 serum dilution/plaque count without addition of serum using FcγR-expressing BHK cell lines.

c Underline indicates positive infection-enhancing activity. Positive infection-enhancing activity is defined as fold-enhancement value greater than cut-off value plus 2 times SD in the mean plaque count in the presence of human serum samples as compared to the cut-off value. Cut-off value was determined in the absence of serum.

d Serum samples obtained from selected non acute dengue patients.

Interestingly, serum samples from patients with acute secondary infection (#46, 49, 56, 40, 42, 43, 44, 45, 52, and 54) demonstrated neutralizing antibody titers of 1∶10–1∶80 to the infecting serotype as determined using BHK cells, but these serum samples showed no neutralizing antibody titers to the infecting serotype as determined using FcγR-expressing BHK cells (Table 3). The results indicate that neutralizing antibody titers determined by using FcγR-expressing BHK cells were lower than those determined by using FcγR-negative BHK cells, and that for some of the samples, neutralizing antibody titers were detected only by using FcγR-negative BHK cells, but not by using FcγR-expressing BHK cells. Major target cells of DENV in vivo are FcγR-expressing cells such as monocyte-lineage cells [34]–[37]. The results suggest that the titers determined by using FcγR-expressing BHK cells may, thus, reflect actual biological activities of antibodies in vivo.

### Enhancing activity to 4 DENV serotypes in serum samples with neutralizing activity

Fold-enhancement to DENV-1 ranged from 0.7–5.6; DENV-2, <0.1–2.1, DENV-3, 0.9–4.9, and DENV-4, 1.1–7.0 with serum sample from patients with infecting serotype of DENV-1. Fold-enhancement to DENV-1 ranged from <0.1–5.5; DENV-2, <0.1–2.3; DENV-3, 0.6–6.7, and DENV-4, 0.9–7.1 with serum samples from patients with infecting serotype of DENV-3 (Table 4, Table S3). Some of the samples from non acute dengue patients also exhibited enhancing activity to DENV (Table S1).

**Figure 1:**
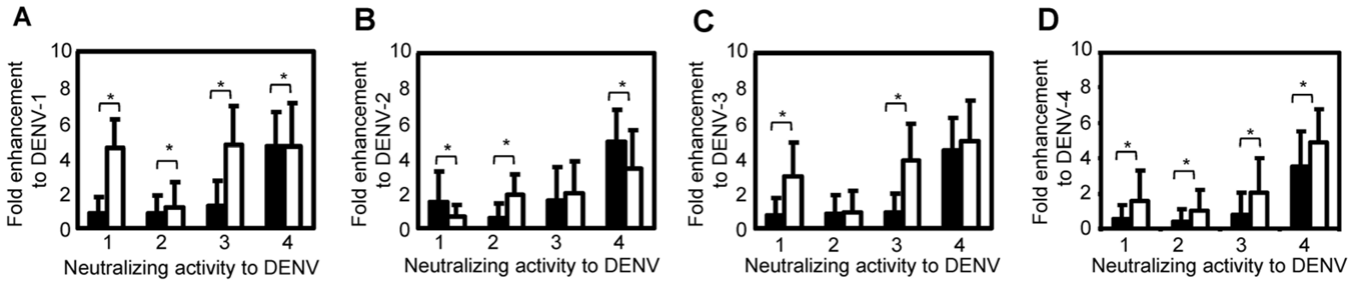
Infection-enhancement activity in serum samples with neutralizing activity to each of the four DENV serotypes. Sixty serum samples exhibiting neutralizing activity to DENV were analyzed for presence of infection-enhancement activity to each of the four DENV serotypes: (A) DENV-1, (B) DENV-2, (C) DENV-3 and, (D) DENV-4. Infection-enhancement activity to each DENV serotype was determined by using FcγR-expressing cells by the formula: (mean plaque count at 1∶10 serum dilution)/(mean plaque count in the absence of human serum samples), and expressed as fold enhancement to each DENV serotype. Closed bars indicate serum samples with neutralizing titer PRNT_50_≥10 to the indicated DENV serotype and open bars indicate serum samples with neutralizing titer PRNT_50_<10 as determined using FcγR-negative BHK cells. (*) indicates P<0.05.

**Figure 2:**
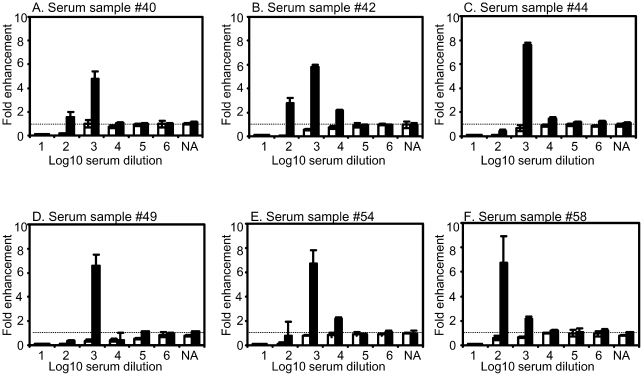
Infection-enhancement activity in serum samples with neutralizing activity to DENV-2. Six serum samples with high neutralizing activity to DENV-2 at 1∶10 serum dilutions were tested for presence of infection-enhancement activity to DENV-2 at serum dilutions of 1∶10 to 1∶10^6^. (A) serum sample #40, (B) #42, (C) #44 (D) #49, (E) #54 and, (F) #58. Infection-enhancement activity to each DENV serotype was determined by using FcγR-expressing BHK cells and BHK cells by the formula: (mean plaque count at each serum dilution)/(mean plaque count in the absence of human serum samples), and expressed as fold enhancement to DENV-2. Closed bars indicate FcγR-expressing BHK cells and open bars indicate FcγR-negative BHK cells.

The results indicate that sera from patients with DENV infection possess the ability to enhance the infection by the infecting serotype. The ability of serum samples with DENV neutralizing activity to enhance each of the 4 DENV serotypes at 1∶10 serum dilution was analyzed (Figure 1). Serum samples with neutralizing titers of ≥1∶10 determined by using BHK cells demonstrated lower levels of fold-enhancement to the homotypic serotypes. Fold enhancement to DENV-1 of serum samples with DENV-1 neutralizing antibody (NA) titer of ≥1∶10 was 0.8±0.9 versus DENV-1 NA titer <1∶10 = 4.5±1.6 (P<0.01). Fold infection-enhancement to DENV-2 using serum samples with DENV-2 neutralizing antibody (NA) titers of ≥1∶10 was 0.6±0.9, while that of samples with DENV-2 NA titers <1∶10 was 1.9±1.1 (P<0.01). Fold enhancement to DENV-3 of serum samples with DENV-3 neutralizing antibody (NA) titer of ≥1∶10 was 0.9±1.1, while DENV-3 NA titers <1∶10 = 3.9±2.1 (P<0.01). Fold enhancement to DENV-4 of serum samples with DENV-4 neutralizing antibody (NA) titer of ≥1∶10 was 3.6±2.0, while that of samples with DENV-4 NA titers <1∶10 was 4.9±1.9 (P = 0.02) (Figure 1). Serum samples with high levels of neutralizing activity to DENV-2 (40, 42, 44, 49, 54 and 58) exhibited peak fold enhancement ranging from 5.8–7.6 at higher serum dilutions of 1∶100–1∶1000 (Figure 2).

Of the 55 serum samples from non acute dengue patients, 42 exhibited infection-enhancement activity to DENV (Table S1). Enhancement activities against DENV-1 and DENV-3 using serum samples obtained from 7 patients with acute DENV-1 infection (mean fold-enhancement = 3.1, P = 0.02, and mean fold-enhancement = 3.2, P = 0.02 respectively) were significantly higher than to those of samples from non-acute DENV patients with multitypic neutralizing activity to ≥3 DENV serotypes (mean fold-enhancement = 0.6 to DENV-1, mean fold-enhancement = 0.8 to DENV-3). Similarly, enhancement activities against DENV-3 of samples from 11 patients with acute DENV-3 infection (mean fold-enhancement = 2.4, P = 0.04) were higher than those of samples from non acute DENV patients with multitypic neutralizing activity to ≥3 DENV serotypes (Table S5).

Using serum samples at 1∶10 dilution, neutralizing activities were higher in samples from patients with multitypic neutralizing activity to ≥3 DENV serotypes (mean percentage of DENV-1 plaque reduction = 92%, DENV-3 plaque reduction = 92%) than in those from patients with acute secondary DENV-1 infection (DENV-1 plaque reduction = 43%, P<0.01; DENV-3 plaque reduction = 34%, P<0.01) and, than in those from patients with acute secondary DENV-3 infection (DENV-1 plaque reduction = 63%, P = 0.02; DENV-3 plaque reduction = 55%, P<0.01) (Table S6).

Neutralizing titers were higher in samples from non acute dengue patients with neutralizing activity to multitypic DENV than in those from patients with acute primary and secondary DENV infection. In contrast, enhancing activity was significantly lower in non acute dengue patients with neutralizing activity to multitypic DENV than in those from patients with acute secondary DENV infection (Table S5). The results suggest that serum samples with high neutralizing activity possess no or only low levels of infection-enhancing activity to respective serotypes at low serum dilutions. In contrast, serum samples without neutralizing activity possess high levels of enhancing activity.

## Discussion

The relationship between neutralizing activity determined using FcγR-negative cells and infection-enhancing activity determined using FcγR-expressing cells was examined in the present study. We determined that a high proportion of the serum samples from residents of a DENV endemic country, Malaysia, possessed DENV neutralizing and infection-enhancing activity (75%, 60/80; Table 2). The neutralizing antibody titers were higher when determined by using FcγR-negative cells than when determined by using FcγR-expressing cells (Table 3). Mammalian cells such as Vero cells and BHK cells are commonly used in plaque reduction neutralizing tests [39]. However, in the absence of FcγR, these cells exclusively detect neutralizing antibody titers. Other investigators have suggested that the use of FcγR-expressing cells, including monocyte-lineage THP-1 cells and K562 cells, dendritic cells, FcγR-expressing CV-1 and BHK cells, and, DC-SIGN expressing RAJI cells and U937 cells may better reflect the in vivo neutralizing titers [40]–[46]. Surrogate plaque titration assays are, however, required to determine virus titers in non-adherent monocyte-lineage cells. Infection-enhancement was also detected using an FcγR-expressing cell line, THP-1 cells, for a subset of serum samples (#39, 40, 28 and 77) which exhibited high infection-enhancement activity to DENV-4 using FcγR-expressing BHK cells (data not shown). Thus, the results of ADE assays were consistent between THP-1 cells and FcγR-expressing BHK. In addition, serum samples (#46, 40, 42, 43, 44, 45, 52 and 54) that demonstrated neutralizing antibody titers to DENV-2 (1∶10 to >1∶1280) along with neutralizing activities to other serotypes in FcγR-negative BHK cells exhibited neutralizing antibody only to DENV-2 in FcγR-expressing BHK cells. Cross-reactive neutralizing activity determined using FcγR-negative cells was not detected using FcγR-positive cells. This could be due to hampered neutralizing activity to heterologous serotypes by infection-enhancing activity [25], [42], [47]. Importantly, our results suggest that the FcγR-expressing BHK cells provides more informative data on serotype-specific neutralizing activity, and may better reflect protection in vivo.

Presence of infection-enhancing activity in antibodies with neutralizing activity to the infecting serotype has been reported previously [37], [38], [47]. Interestingly, despite the result that 11 serum samples from patients with DENV infection exhibited neutralizing activity to the infecting serotype at 1∶10–1∶80 as determined by using FcγR-negative BHK cells, the neutralizing titer to the respective infecting serotypes as determined by using FcγR-expressing BHK cells was <1∶10 in all of the 11 serum samples tested. It has been reported that the main target cells of DENV infection in vivo are FcγR-expressing cells, such as monocytes and macrophages [34]–[36]. The results suggest that DENV-antibody complexes which are incapable of infecting FcγR-negative cells, may retain the ability to infect FcγR-expressing cells due to the presence of FcγR. However, further studies are required to identify the relationship between infection-enhancing activity and neutralizing activity during infection in vivo.

All four DENV serotypes are found co-circulating in Malaysia. Although data was not available for the pre-dominant dengue virus serotype in Perak prior to 2008, DENV-2 was the dominant circulating serotype in peninsular Malaysia between 1998–2000 and 2006–2007 (Chua et al., unpublished data). High levels of neutralizing activity against DENV-2 were detected in some of the serum samples (Table 3), indicate that these individuals may have been previously exposed to DENV-2. Previous studies have suggested that ADE activity influences disease severity in patients with secondary DENV infection [32], [48]. As low levels of serum dilutions may better reflect in vivo conditions, serum samples at dilutions of 1∶10 were used in the present study. The results showed infection-enhancing activity (fold enhancement) of 0.7–6.7 to the infecting serotype at serum dilutions of 1∶10. The assay using FcγR-expressing BHK cells detected infection-enhancing activity to infecting serotypes at low serum dilutions in serum samples with neutralizing activity, suggesting the presence of ADE activity in vivo. Interestingly, there was an inverse relationship between infection-enhancement activity to a DENV serotype and high neutralizing activity as determined by using FcγR-negative BHK cells to the respective serotypes (Figure 2, Table 3, Table 4).

Previous studies showed that higher dilutions of patient serum samples, in quantities that are not sufficient to support neutralization, enhance DENV infection [32], [47]. Patient serum samples #49, 58, 42, 45, and 54, require higher concentrations for neutralization in the presence of FcγR than in the absence of FcγR. In addition, serum samples #57, 23, 28, 30, 74, 77, and 78 exhibited similar neutralizing antibody titers in both FcγR-expressing BHK cells and FcγR-negative BHK cells [25]. Some antibodies may require lower threshold occupancy for neutralization, and, thus, virus neutralization may occur at similar concentrations both in FcγR-negative and FcγR-expressing cells. Alternatively, binding of some antibodies may lead to DENV conformational changes [49], and therefore, result in virus neutralization both in the presence and absence of FcγR, at similar antibody concentrations. The FcγR-expressing BHK cell-based assay system is unique as antibody neutralizing activity could be analyzed and compared simultaneously using one cell line (BHK cell line) either in the absence or presence of FcγR by a conventional plaque assay.

It is known that primary infection with one DENV serotype induces long-term protection to infection with the same serotype [50]. By using serum samples from a DENV-endemic area, we demonstrated that infection-enhancing activity which was determined only by using FcγR-expressing cells hampers neutralizing activity that was determined using FcγR-negative BHK cells. Moreover, infection-enhancing activity was also detected in serum samples with low or negative neutralizing activity that were determined using FcγR-negative BHK cells. Although in vitro systems may not faithfully reflect all aspects of DENV infection in vivo, the results suggest that as compared to the neutralizing activity determined by FcγR-negative cell culture system, the sum of neutralizing and enhancing activity determined by the FcγR-expressing cells may better reflect protection to DENV infection in vivo. Further studies are, however, needed to define the relationship between protection and neutralizing titers determined by using FcγR-positive cells.

## Supporting Information

Table S1 Levels of neutralizing and infection-enhancing activity in serum samples obtained from 42 non-acute dengue patients against each of the four dengue virus serotypes.
(DOC)

Table S2 Absence of neutralizing and infection-enhancing activities of serum samples obtained from 13 non-acute dengue patients against each of the four dengue virus serotypes.
(DOC)

Table S3 Levels of neutralizing and infection-enhancing activities of serum samples obtained from seven DENV-1 patients and eleven DENV-3 patients against each of the four dengue virus serotypes.
(DOC)

Table S4 Absence of neutralizing and infection-enhancing activity in serum samples obtained from 5 acute primary DENV-1 dengue patients and 2 acute primary DENV-3 patients against each of the four dengue virus serotypes.
(DOC)

Table S5 Levels of enhancement activity against four DENV serotypes in serum samples from patients with acute DENV-1 and DENV-3 infection in comparison with those in samples from patients with non acute DENV infection at 1∶10 serum dilution as determined by using FcγR-expressing BHK cells.
(DOC)

Table S6 Levels of neutralizing activity against four DENV serotypes in serum samples from patients with acute DENV-1 and DENV-3 infection in comparison to those from patients with non-acute DENV infection, at 1∶10 dilution, as determined by using FcγR-negative BHK cells.
(DOC)

## References

[1] World Health Organization (2009). Dengue: Guidelines for diagnosis, treatment, prevention and control.. http://whqlibdoc.who.int/publications/2009/9789241547871_eng.pdf.

[2] Srikiatkhachorn A, Gibbons RV, Green S, Libraty DH, Thomas SJ (2010). Dengue hemorrhagic fever: the sensitivity and specificity of the world health organization definition for identification of severe cases of dengue in Thailand, 1994–2005.. Clin Infect Dis.

[3] Barrera R, Delgado N, Jiménez M, Valero S (2002). Eco-epidemiological factors associated with hyperendemic dengue haemorrhagic fever in Maracay City, Venezuela.. Dengue Bull.

[4] Lai PC, Lee SS, Kao CH, Chen YS, Huang CK (2004). Characteristics of a dengue hemorrhagic fever outbreak in 2001 in Kaohsiung.. J Microbiol Immunol Infect.

[5] Dengue Net-WHO Internet-based system for global surveillance of dengue fever and dengue haemorrhagic fever (dengue/DHF).. http://apps.who.int/globalatlas/default.asp.

[6] Department of Health, Malaysia (2011). (In Malay) Press release: Dengue fever and chikungunya.. http://www.moh.gov.my/pr_categories/1/press_releases.

[7] Abu Bakar S, Wong PF, Chan YF (2002). Emergence of dengue virus type 4 genotype IIA in Malaysia.. J Gen Virol.

[8] Wong SS, Abdul Jamil J, Abu Bakar S (2007). Antibody neutralization and viral virulence in recurring dengue virus type 2 outbreaks.. Viral Immunol.

[9] Fan WF, Yu SR, Cosgriff TM (1989). The reemergence of dengue in China.. Rev Infect Dis.

[10] Shu PY, Chien LJ, Chang SF, Su CL, Kuo YC (2005). Fever screening at airports and imported dengue.. Emerg Infect Dis.

[11] Zheng K, Zhou HQ, Yan J, Ke CW, Maeda A (2009). Molecular characterization of the E gene of dengue virus type 1 isolated in Guangdong province, China, in 2006.. Epidemiol Infect.

[12] Guzmán MG, Kouri G, Valdes L, Bravo J, Alvarez M (2000). Epidemiologic studies on dengue in Santiago de Cuba, 1997.. Am J Epidemiol.

[13] Kosasih H, Yusuf H, Sudjana P, Alisjahbana B, Wuryadi S (2006). Report of four volunteers with primary, secondary and tertiary dengue infections during a prospective cohort study.. Dengue Bull.

[14] Sangkawibha N, Rojanasuphot S, Ahandrik S, Viriyapongse S, Jatanasen S (1984). Risk factors in dengue shock syndrome: a prospective epidemiologic study in Rayong, Thailand. I. The 1980 outbreak.. Am J Epidemiol.

[15] Alvarez M, Rodriguez-Roche R, Bernardo L, Vázquez S, Morier L (2006). Dengue hemorrhagic fever caused by sequential dengue 1–3 virus infections over a long time interval: Havana epidemic, 2001–2002.. Am J Trop Med Hyg.

[16] Tee HP, How SH, Jamalludin AR, Safhan MN, Sapian MM (2009). Risk factors associated with development of dengue haemorrhagic fever or dengue shock syndrome in adults in Hospital Tengku Ampuan Afzan Kuantan.. Med J Malaysia.

[17] Fried JR, Gibbons RV, Kalayanarooj S, Thomas SJ, Srikiatkhachorn A (2010). Serotype-specific differences in the risk of dengue hemorrhagic fever: an analysis of data collected in Bangkok, Thailand from 1994 to 2006.. Plos Negl Trop Dis.

[18] Modhiran N, Kalayanarooj S, Ubol S (2010). Subversion of innate defenses by the interplay between DENV and pre-existing enhancing antibodies: TLRs signaling collapse.. PLoS Negl Trop Dis.

[19] Ubol S, Phuklia W, Kalayanarooj S, Modhiran N (2010). Mechanisms of immune evasion induced by a complex of dengue virus and preexisting enhancing antibodies.. J Infect Dis.

[20] Midgley CM, Bajwa-Joseph M, Vasanawathana S, Limpitikul W, Wills B (2011). An in-depth analysis of original antigenic sin in dengue virus infection.. J Virol.

[21] Moi ML, Lim CK, Kotaki A, Takasaki T, Kurane I (2011). Detection of higher levels of dengue viremia using FcγR-expressing BHK-21 cells than FcγR-negative cells in secondary Infection but not in primary infection.. J Infect Dis.

[22] Klomporn P, Panyasrivanit M, Wikan N, Smith DR (2011). Dengue infection of monocytic cells activates ER stress pathways, but apoptosis is induced through both extrinsic and intrinsic pathways.. Virology.

[23] Kou Z, Lim JY, Beltramello M, Quinn M, Chen H (2011). Human antibodies against dengue enhance dengue viral infectivity without suppressing type I interferon secretion in primary human monocytes.. Virology.

[24] Libraty DH, Acosta LP, Tallo V, Segubre-Mercado E, Bautista A (2009). A prospective nested case-control study of dengue in infants: rethinking and refining the antibody-dependent enhancement dengue hemorrhagic fever model.. PloS Med.

[25] Moi ML, Lim CK, Kotaki A, Takasaki T, Kurane I (2010). Discrepancy in neutralizing antibody titers between plaque reduction neutralizing test using FcγR-negative and FcγR-expressing BHK cells.. Clin Vac Immunol.

[26] Cardosa MJ, Choo BH, Zuraini I (1992). A serological study of Japanese encephalitis virus infections in northern Peninsular Malaysia.. Southeast Asian J Trop Med Public Health.

[27] Nayar SK, Noridah O, Paranthaman V, Ranjit K, Norizah I (2007). Co-infection of dengue virus and chikungunya virus in two patients with acute febrile illness.. Med J Malaysia.

[28] Chin PS, Khoo AP, Asmah Hani AW, Chem YK, Norizah I (2008). Acute dengue in a neonate secondary to perinatal transmission.. Med J Malaysia.

[29] Ito M, Takasaki T, Yamada K, Nerome R, Tajima S (2004). Development and evaluation of fluorogenic taqman reverse transcriptase PCR assays for detection of dengue virus types 1 to 4.. J Clin Microbiol.

[30] Moi ML, Takasaki T, Kotaki A, Tajima S, Lim CK (2010). Importation of dengue virus type 3 to Japan from Tanzania and Côte d'Ivoire.. Emerg Infect Dis.

[31] Roehrig J (2007). Guidelines for plaque reduction neutralizing testing of human antibodies to dengue viruses.. http://whqlibdoc.who.int/hq/2007/who_ivb_07.07_eng.pdf.

[32] Kliks S, Nisalak A, Brandt WE, Wahl L, Burke DS (1989). Antibody dependent enhancement of dengue virus growth in human monocytes as risk factor for dengue hemorrhagic fever.. Am J Trop Med Hyg.

[33] Konishi E, Tabuchi Y, Yamanaka A (2010). A simple assay system for infection-enhancing and -neutralizing antibodies to dengue type 2 virus using layers of semi-adherent K562 cells.. J Virol Methods.

[34] Blackley S, Kou Z, Chen H, Quinn M, Rose RC (2007). Primary human splenic macrophages, but not T or B cells, are the principal target cells for dengue virus infection in vitro..

[35] Kou Z, Quinn M, Chen H, Rodrigo WW, Rose RC (2008). Monocytes, but not T or B cells, are the principal target cells for dengue virus (DV) infection among human peripheral blood mononuclear cells.. J Med Virol.

[36] Boonnak K, Dambach KM, Donofrio GC, Tassaneetrithep B, Marovich MA (2011). Cell type specificity and host genetic polymorphisms influence antibody-dependent enhancement of dengue virus infection.. J Virol.

[37] Laoprasopwattana K, Libraty DH, Endy TP, Nisalak A, Chunsuttiwat S (2004). Dengue Virus (DV) enhancing antibody activity in preillness plasma does not predict subsequent disease severity or viremia in secondary DV infection.. J Infect Dis.

[38] Hatch S, Endy TP, Thomas S, Mathew A, Potts J (2011). Intracellular Cytokine Production by Dengue Virus-specific T cells Correlates with Subclinical Secondary Infection.. J Infect Dis.

[39] World Health Organization (2007). Guidelines for plaque reduction neutralization testing of human antibodies to dengue viruses.. http://whqlibdoc.who.int/hq/2007/who_ivb_07.07_eng.pdf.

[40] Tassaneetrithep B, Burgess TH, Granelli-Piperno A, Trumpfheller C, Finke J (2003). DC-SIGN (CD209) mediates dengue virus infection of human dendritic cells.. J Exp Med.

[41] Rodrigo WW, Jin X, Blackley SD, Rose RC, Schlesinger JJ (2006). Differential enhancement of dengue virus immune complex infectivity mediated by signaling-competent and signaling-incompetent human Fcgamma RIA (CD64) or FcgammaRIIA (CD32).. J Virol.

[42] Rodrigo WW, Alcena DC, Kou Z, Kochel TJ, Porter KR (2009). Difference between the abilities of human Fcgamma receptor-expressing CV-1 cells to neutralize American and Asian genotypes of dengue virus 2.. Clin Vac Immunol.

[43] Shanaka WW, Rodrigo I, Alcena DC, Rose RC, Jin X (2009). An automated Dengue virus microneutralization plaque assay performed in human Fc(gamma) receptor-expressing CV-1 cells.. Am J Trop Med Hyg.

[44] Martin NC, Pardo J, Simmons M, Tjaden JA, Widjaja S (2006). An immunocytometric assay based on dengue infection via DC-SIGN permits rapid measurement of anti-dengue neutralizing antibodies.. J Virol Methods.

[45] Kraus Messer W, Haymore LB, de Silva AM (2007). Comparison of plaque- and flow cytometry-based methods for measuring dengue virus neutralization.. J Clin Microbiol.

[46] Boonnak K, Slike BM, Burgess TH, Mason RM, Wu SJ (2008). Role of dendritic cells in antibody-dependent enhancement of dengue virus infection.. J Virol.

[47] Morens DM, Larsen LK, Halstead SB (1987). Study of the distribution of antibody-dependent enhancement determinants on dengue 2 isolates using dengue 2-derived monoclonal antibodies.. J Med Virol.

[48] Endy TP, Nisalak A, Chunsuttitwat S, Vaughn DW, Green S (2004). Relationship of preexisting dengue virus (DV) neutralizing antibody levels to viremia and severity of disease in a prospective cohort study of DV infection in Thailand.. J Infect Dis.

[49] Lok SM, Kostyuchenko V, Nybakken GE, Holdaway HA, Battisti AJ (2008). Binding of a neutralizing antibody to dengue virus alters the arrangement of surface glycoproteins.. Nat Struct Mol Biol.

[50] Sabin AB (1952). Research on dengue fever during World War II.. Am J Trop Med Hyg.

