# Supplementary material for: Dengue Virus Infection-Enhancing Activity in Serum Samples with Neutralizing Activity as Determined by Using FcγR-Expressing Cells
Source: PLoS Negl Trop Dis. 2012 Feb 28;6(2):e1536. doi: 10.1371/journal.pntd.0001536 (PMC3289619; doi:10.1371/journal.pntd.0001536)
Supplement: Table S1 — Levels of neutralizing and infection-enhancing activity in serum samples obtained from 42 non-acute dengue patients against each of the four dengue virus serotypes. (DOC) [file pntd.0001536.s001.doc]

Table S1. Levels of neutralizing and infection-enhancing activity in serum samples obtained from 42 non-acute dengue patients against each of the four dengue virus serotypes.

| Patient no. | DENV-1 | | DENV-2 | | DENV-3 | | DENV-4 | |
| --- | --- | --- | --- | --- | --- | --- | --- | --- |
|  | % Plaque reduction^a^ | Fold enhancement^b^ | % Plaque reduction | Fold enhancement | % Plaque reduction | Fold enhancement | % Plaque reduction | Fold enhancement |
| 1 | 83 | 0.8 | 22 | 4.1 | 10 | 4.2 | 12 | 6.1 |
| 2 | 100 | <0.1 | 48 | 1.4 | 100 | <0.1 | 9 | 3.0 |
| 3 | 85 | 1.5 | 100 | <0.1 | 81 | 1.2 | 35 | 6.4 |
| 4 | 100 | 0.1 | 100 | 0.1 | 94 | <0.1 | 88 | 2.2 |
| 5 | 83 | 1.0 | 100 | <0.1 | 97 | 1.0 | 85 | 2.7 |
| 7 | 100 | <0.1 | 48 | 2.7 | 97 | 1.8 | 12 | 5.5 |
| 8 | 61 | 3.0 | 100 | <0.1 | 100 | <0.1 | 6 | 5.9 |
| 9 | 88 | 2.5 | 26 | 2.8 | 32 | 5.7 | 12 | 6.4 |
| 11 | 100 | <0.1 | 78 | 0.9 | 77 | 0.8 | 3 | 4.8 |
| 12 | 83 | 1.3 | 100 | <0.1 | 48 | 1.1 | 32 | 6.4 |
| 13 | 44 | 3.9 | 52 | 3.9 | 77 | 0.6 | 6 | 4.5 |
| 14 | 78 | 1.4 | 100 | <0.1 | 65 | 4.0 | 41 | 6.0 |
| 15 | 95 | 1.0 | 0 | 4.1 | 58 | 4.3 | 18 | 5.4 |
| 16 | 98 | 0.3 | 100 | 0.1 | 100 | 0.1 | 91 | 5.1 |
| 17 | 100 | <0.1 | 100 | <0.1 | 100 | <0.1 | 53 | 1.6 |
| 18 | 100 | 0.3 | 87 | 0.9 | 84 | 1.6 | 41 | 5.0 |
| 20 | 61 | 1.2 | 100 | <0.1 | 45 | 1.6 | 15 | 5.2 |
| 21 | 100 | <0.1 | 65 | 1.5 | 87 | 0.8 | 12 | 4.9 |
| 23 | 80 | 2.5 | 100 | <0.1 | 87 | 1.3 | 18 | 4.4 |
| 24 | 100 | <0.1 | 100 | <0.1 | 97 | <0.1 | 100 | 0.3 |
| 26 | 100 | <0.1 | 52 | 2.2 | 100 | <0.1 | 41 | 6.2 |
| 28 | 100 | 0.2 | 30 | 2.4 | 90 | 4.1 | 59 | 5.5 |
| 29 | 98 | 0.7 | 91 | 0.6 | 100 | 2.1 | 56 | 6.2 |
| 30 | 59 | 3.2 | 100 | 0.1 | 77 | 2.5 | 56 | 6.1 |
| 34 | 83 | 0.3 | 30 | 0.6 | 55 | 0.8 | 9 | 0.8 |
| 35 | 80 | 1.2 | 39 | 1.2 | 100 | <0.1 | 9 | 0.9 |
| Table S1 (continued) | | | | | | | | |
| Patient no. | DENV-1 | | DENV-2 | | DENV-3 | | DENV-4 | |
|  | % Plaque reduction^a^ | Fold enhancement^b^ | % Plaque reduction | Fold enhancement | % Plaque reduction | Fold enhancement | % Plaque reduction | Fold enhancement |
| 36 | 100 | <0.1 | 95 | 1.1 | 100 | 0.4 | 15 | 6.3 |
| 37 | 5 | 0.9 | 9 | 1.0 | 0 | 3.2 | 12 | 1.0 |
| 38 | 32 | 5.1 | 100 | <0.1 | 26 | 6.3 | 3 | 5.6 |
| 64 | 100 | <0.1 | 74 | 1.6 | 100 | <0.1 | 18 | 4.6 |
| 65 | 100 | <0.1 | 100 | <0.1 | 100 | <0.1 | 74 | 3.6 |
| 66 | 100 | 0.3 | 100 | <0.1 | 100 | <0.1 | 32 | 5.0 |
| 70 | 100 | <0.1 | 91 | 1.2 | 100 | 0.6 | 62 | 4.4 |
| 71 | 100 | <0.1 | 100 | <0.1 | 100 | 0.1 | 59 | 5.1 |
| 72 | 100 | <0.1 | 96 | 0.5 | 97 | 0.4 | 44 | 2.0 |
| 73 | 98 | <0.1 | 48 | 1.2 | 71 | 1.1 | 38 | 2.5 |
| 74 | 98 | <0.1 | 48 | 1.0 | 100 | 0.1 | 74 | 3.9 |
| 75 | 100 | <0.1 | 87 | 1.1 | 94 | 0.2 | 44 | 6.1 |
| 76 | 61 | 3.0 | 91 | <0.1 | 45 | 2.1 | 12 | 5.3 |
| 77 | 100 | <0.1 | 61 | 1.9 | 74 | 1.3 | 9 | 6.7 |
| 78 | 66 | 1.7 | 100 | <0.1 | 55 | 1.3 | 15 | 4.7 |
| 79 | 73 | 1.8 | 61 | 1.4 | 100 | <0.1 | 12 | 6.6 |

^a^ Percentage (%) of plaque reduction to four dengue serotypes was determined using 1:10 diluted serum samples by using FcγR negative BHK cells.

^b^ Fold enhancement was calculated by the formula: number of plaques in the presence of 1:10 diluted serum/ number of plaques in the absence of serum, by using FcγR-expressing BHK cells.
