# Supplementary material for: Dengue Virus Infection-Enhancing Activity in Serum Samples with Neutralizing Activity as Determined by Using FcγR-Expressing Cells
Source: PLoS Negl Trop Dis. 2012 Feb 28;6(2):e1536. doi: 10.1371/journal.pntd.0001536 (PMC3289619; doi:10.1371/journal.pntd.0001536)
Supplement: Table S2 — Absence of neutralizing and infection-enhancing activities of serum samples obtained from 13 non-acute dengue patients against each of the four dengue virus serotypes. (DOC) [file pntd.0001536.s002.doc]

Table S2. Absence of neutralizing and infection-enhancing activities of serum samples obtained from 13 non-acute dengue patients against each of the four dengue virus serotypes.

| Patient no. | DENV-1 | | DENV-2 | | DENV-3 | | DENV-4 | |
| --- | --- | --- | --- | --- | --- | --- | --- | --- |
|  | % Plaque reduction^a^ | Fold enhancement^b^ | % Plaque reduction | Fold enhancement | % Plaque reduction | Fold enhancement | % Plaque reduction | Fold enhancement |
| 6 | 0 | 0.9 | 0 | 1.0 | 10 | 1.0 | 21 | 0.9 |
| 10 | 22 | 0.8 | 17 | 0.9 | 19 | 1.1 | 0 | 1.0 |
| 19 | 0 | 1.0 | 0 | 1.1 | 26 | 0.9 | 0 | 1.0 |
| 22 | 5 | 0.9 | 0 | 0.8 | 19 | 1.1 | 9 | 1.0 |
| 25 | 0 | 0.9 | 13 | 0.7 | 0 | 1.1 | 12 | 1.1 |
| 27 | 17 | 0.8 | 0 | 0.8 | 3 | 1.2 | 15 | 1.1 |
| 31 | 5 | 0.9 | 9 | 0.9 | 13 | 1.0 | 15 | 0.9 |
| 32 | 7 | 1.0 | 0 | 1.2 | 3 | 1.3 | 3 | 0.9 |
| 33 | 12 | 0.9 | 9 | 0.8 | 0 | 1.0 | 15 | 0.9 |
| 67 | 0 | 0.8 | 0 | 1.0 | 10 | 1.0 | 12 | 1.0 |
| 68 | 0 | 0.8 | 0 | 0.9 | 29 | 0.9 | 18 | 1.0 |
| 69 | 0 | 0.9 | 4 | 1.2 | 16 | 0.9 | 15 | 1.2 |
| 80 | 15 | 0.9 | 13 | 0.9 | 13 | 0.9 | 3 | 1.0 |

^a^ Percentage (%) of plaque reduction to four dengue serotypes was determined using 1:10 diluted serum samples by using FcγR negative BHK cells.

^b^ Fold enhancement was calculated by the formula: number of plaques in the presence of 1:10 diluted serum/ number of plaques in the absence of serum, by using FcγR-expressing BHK cells.
