# Supplementary material for: Dengue Virus Infection-Enhancing Activity in Serum Samples with Neutralizing Activity as Determined by Using FcγR-Expressing Cells
Source: PLoS Negl Trop Dis. 2012 Feb 28;6(2):e1536. doi: 10.1371/journal.pntd.0001536 (PMC3289619; doi:10.1371/journal.pntd.0001536)
Supplement: Table S3 — Levels of neutralizing and infection-enhancing activities of serum samples obtained from seven DENV-1 patients and eleven DENV-3 patients against each of the four dengue virus serotypes. (DOC) [file pntd.0001536.s003.doc]

Table S3. Levels of neutralizing and infection-enhancing activities of serum samples obtained from seven DENV-1 patients and eleven DENV-3 patients against each of the four dengue virus serotypes.

| Patient no. | DENV-1 | | | DENV-2 | | DENV-3 | | DENV-4 | |
| --- | --- | --- | --- | --- | --- | --- | --- | --- | --- |
|  | % Plaque reduction^a^ | Fold enhancement^b^ | | % Plaque reduction | Fold enhancement | % Plaque reduction | Fold enhancement | % Plaque reduction | Fold enhancement |
| 1. Secondary   DENV-1 infection | | | | | |  |  |  |  |
| 46 | 45 | 0.7 | 100 | | 0.1 | 84 | 0.9 | 74 | 5.6 |
| 47 | 7 | 5.6 | 91 | | 2.1 | 35 | 5.1 | 9 | 1.2 |
| 48 | 32 | 4.7 | 61 | | 2.0 | 13 | 5.3 | 3 | 4.4 |
| 49 | 51 | 1.2 | 100 | | <0.1 | 23 | 1.0 | 3 | 2.0 |
| 56 | 56 | 2.8 | 87 | | 1.6 | 16 | 4.2 | 21 | 6.9 |
| 57 | 83 | 1.2 | 100 | | <0.1 | 68 | 1.3 | 21 | 4.7 |
| 58 | 29 | 5.3 | 100 | | <0.1 | 0 | 4.9 | 9 | 6.0 |
|  |  |  |  | |  |  |  |  |  |
| 1. Secondary   DENV-3 infection | | |  | |  |  |  |  |  |
| 39 | 98 | <0.1 | 35 | | 2.3 | 58 | 1.5 | 26 | 6.1 |
| 40 | 61 | 1.9 | 100 | | <0.1 | 84 | 1.2 | 6 | 4.6 |
| 41 | 12 | 5.5 | 100 | | <0.1 | 23 | 5.3 | 41 | 6.5 |
| 42 | 55 | 0.5 | 100 | | <0.1 | 100 | 1.7 | 76 | 1.4 |
| 43 | 0 | 0.5 | 100 | | 0.9 | 94 | 1.9 | 35 | 5.3 |
| 44 | 59 | 1.5 | 100 | | <0.1 | 48 | 4.6 | 26 | 7.3 |
| 45 | 85 | 0.7 | 100 | | <0.1 | 32 | 0.8 | 35 | 5.9 |
| 52 | 95 | 1.5 | 0 | | <0.1 | 26 | 0.9 | 3 | 0.9 |
| 53 | 95 | 5.2 | 100 | | 0.2 | 71 | 1.4 | 41 | 4.7 |
| 54 | 95 | 0.8 | 100 | | <0.1 | 42 | 2.0 | 61 | 5.0 |
| 55 | 44 | 5.2 | 100 | | 0.2 | 26 | 6.7 | 3 | 6.9 |

^a^ Percentage (%) of plaque reduction to four dengue serotypes were determined at 1:10 dilution by using FcγR negative BHK cells.

^b^ Fold enhancement was calculated by the formula: number of plaques in the presence of 1:10 diluted serum/ number of plaques in the absence of serum, by using FcγR-expressing BHK cells.
