# Supplementary material for: Dengue Virus Infection-Enhancing Activity in Serum Samples with Neutralizing Activity as Determined by Using FcγR-Expressing Cells
Source: PLoS Negl Trop Dis. 2012 Feb 28;6(2):e1536. doi: 10.1371/journal.pntd.0001536 (PMC3289619; doi:10.1371/journal.pntd.0001536)
Supplement: Table S4 — Absence of neutralizing and infection-enhancing activity in serum samples obtained from 5 acute primary DENV-1 dengue patients and 2 acute primary DENV-3 patients against each of the four dengue virus serotypes. (DOC) [file pntd.0001536.s004.doc]

Table S4. Absence of neutralizing and infection-enhancing activity in serum samples obtained from 5 acute primary DENV-1 dengue patients and 2 acute primary DENV-3 patients against each of the four dengue virus serotypes.

| Patient no. | DENV-1 | | DENV-2 | | | DENV-3 | | DENV-4 | |
| --- | --- | --- | --- | --- | --- | --- | --- | --- | --- |
|  | % Plaque reduction^a^ | Fold enhancement^b^ | | % Plaque reduction | Fold enhancement | % Plaque reduction | Fold enhancement | % Plaque reduction | Fold enhancement |
|  | |  | |  |  |  |  |  |  |
| 1. Primary   DENV-1 infection | |  | |  |  |  |  |  |  |
| 59 | 0 | 1.5 | | 0 | 1.1 | 6 | 1.0 | 32 | 1.1 |
| 60 | 13 | 1.0 | | 13 | 0.8 | 19 | 1.1 | 24 | 1.2 |
| 61 | 32 | 1.0 | | 30 | 1.0 | 16 | 1.0 | 3 | 1.0 |
| 62 | 29 | 0.7 | | 52 | 0.8 | 6 | 1.2 | 41 | 1.0 |
| 63 | 25 | 0.9 | | 17 | 0.8 | 13 | 1.1 | 32 | 1.0 |
|  | |  | |  |  |  |  |  |  |
| 1. Primary   DENV-3 infection | |  | |  |  |  |  |  |  |
| 50 | 0 | 0.9 | | 0 | 1.0 | 32 | 1.2 | 18 | 1.4 |
| 51 | 37 | 0.9 | | 0 | 0.8 | 32 | 1.1 | 0 | 1.0 |

^a^ Percentage (%) of plaque reduction to four dengue serotypes was determined using 1:10 diluted serum samples by using FcγR negative BHK cells.

^b^ Fold enhancement was calculated by the formula: number of plaques in the presence of 1:10 diluted serum/ number of plaques in the absence of serum, by using FcγR-expressing BHK cells.
