# Supplementary material for: Dengue Virus Infection-Enhancing Activity in Serum Samples with Neutralizing Activity as Determined by Using FcγR-Expressing Cells
Source: PLoS Negl Trop Dis. 2012 Feb 28;6(2):e1536. doi: 10.1371/journal.pntd.0001536 (PMC3289619; doi:10.1371/journal.pntd.0001536)
Supplement: Table S5 — Levels of enhancement activity against four DENV serotypes in serum samples from patients with acute DENV-1 and DENV-3 infection in comparison with those in samples from patients with non acute DENV infection at 1∶10 serum dilution as determined by using FcγR-expressing BHK cells. (DOC) [file pntd.0001536.s005.doc]

Table S5. Levels of enhancement activity against four DENV serotypes in serum samples from patients with acute DENV-1 and DENV-3 infection in comparison with those in samples from patients with non acute DENV infection at 1:10 serum dilution as determined by using FcγR-expressing BHK cells.

| Patient | Fold infection-enhancement value to DENV serotype | | | | | | | |
| --- | --- | --- | --- | --- | --- | --- | --- | --- |
|  | DENV-1 | | DENV-2 | | DENV-3 | | DENV-4 | |
|  | Fold^a^ | P^b^ | Fold | P | Fold | P | Fold | P |
|  |  |  |  |  |  |  |  |  |
| (I) Acute primary DENV infection |  |  |  |  |  |  |  |  |
| DENV-1 infection (N=5) | 1.0 (0.7-1.3) | 0.08 | 0.9 (0.8-1.1) | 0.48 | 1.1 (1.0-1.2) | 0.26 | 1.1 (1.0-1.2) | <0.01 |
| DENV-3 infection (N=2) | 0.9 (0.9) | 0.16 | 0.9 (0.8-1.0) | 0.49 | 1.1 (1.1-1.2) | 0.16 | 1.2 (1.0-1.3) | <0.01 |
|  |  |  |  |  |  |  |  |  |
| (II) Acute secondary DENV infection |  |  |  |  |  |  |  |  |
| DENV-1 infection (N=7) | 3.1 (0.7-5.3) | 0.02^d^ | 0.8 (<0.1-2.1) | 0.89 | 3.2 (0.9-5.3) | 0.02 | 4.4 (1.2-6.9) | 0.83 |
| DENV-3 infection (N=11) | 1.8 (<0.1-5.5) | 0.07 | 0.4 (<0.1-2.3) | 0.19 | 2.4 (0.8-6.7) | 0.04 | 5.0 (0.9-7.3) | 0.59 |
|  |  |  |  |  |  |  |  |  |
| (III) Non-acute DENV infection |  |  |  |  |  |  |  |  |
| Absence of neutralizing activity to all serotype (N=13) | 0.9 (0.8-1.0) | 0.14 | 0.9 (0.7-1.2) | 0.32 | 1.2 (0.9-1.2) | 0.19 | 1.0 (0.9-1.2) | <0.01 |
| Neutralizing activity to 1 DENV serotype (N=4^e^) | 2.8 (0.8-2.5) | 0.22 | 2.3 (<0.1-4.1) | 0.33 | 5.4 (3.2-6.3) | <0.01 | 6.0 (1.0-6.4) | <0.01 |
| Neutralizing activity to 2 DENV serotype (N=7) | 1.5 (0.1-3.9) | 0.14 | 1.4 (<0.1-4.1) | 0.40 | 1.7 (0.6-4.2) | 0.14 | 4.3 (0.8-6.4) | 0.73 |
| Neutralizing activity to ≥3 DENV serotype (N=31) | 0.6 (<0.1-3.2) | -^c^ | 0.8 (<0.1-2.7) | - | 0.8 (<0.1-4.1) | - | 4.6 (0.3-6.6) | - |
|  |  |  |  |  |  |  |  |  |

^a^ Mean fold enhancement was calculated by the formula [total (number of plaques in the presence of 1:10 diluted serum/ number of plaques in the absence of serum)/ number of patients] by using FcγR-expressing BHK cells. Values in brackets indicate the lowest and highest fold enhancement values.

^b^ P-value of fold-enhancement compared with those with neutralizing activity to ≥3 DENV serotype.

^c^ Indicates that calculation is not possible.

^d^ Underline indicates P-value of less than 0.05.

^e^ Serum sample #37 does not possess neutralizing activity to any of the 4 DENV serotypes, but exhibited infection-enhancement activity to DENV-3.
