# Supplementary material for: Dengue Virus Infection-Enhancing Activity in Serum Samples with Neutralizing Activity as Determined by Using FcγR-Expressing Cells
Source: PLoS Negl Trop Dis. 2012 Feb 28;6(2):e1536. doi: 10.1371/journal.pntd.0001536 (PMC3289619; doi:10.1371/journal.pntd.0001536)
Supplement: Table S6 — Levels of neutralizing activity against four DENV serotypes in serum samples from patients with acute DENV-1 and DENV-3 infection in comparison to those from patients with non-acute DENV infection, at 1∶10 dilution, as determined by using FcγR-negative BHK cells. (DOC) [file pntd.0001536.s006.doc]

Table S6. Levels of neutralizing activity against four DENV serotypes in serum samples from patients with acute DENV-1 and DENV-3 infection in comparison to those from patients with non-acute DENV infection, at 1:10 dilution, as determined by using FcγR-negative BHK cells.

| Patient | Mean plaque reduction (%) to DENV serotype | | | | | | | | | | | |
| --- | --- | --- | --- | --- | --- | --- | --- | --- | --- | --- | --- | --- |
|  | DENV-1 | | DENV-2 | | DENV-3 | | | | DENV-4 | | | |
|  | %^a^ | P^b^ | % | P | % | | P | | % | | P | |
|  |  |  |  |  |  | |  | |  | |  | |
| (I) Acute primary DENV infection |  |  |  |  |  | |  | |  | |  | |
| DENV-1 infection (N=5) | 20 (0-32) | <0.01 | 22 (0-48) | <0.01 | 12 (6-19) | | <0.01 | | 26 (3-32) | | 0.02 | |
| DENV-3 infection (N=2) | 18 (0-16) | 0.04 | 0 (0) | <0.01 | 32 (32) | | <0.01 | | 9 (0-18) | | <0.01 | |
|  |  |  |  |  |  | |  | |  | |  | |
| (II) Acute secondary DENV infection |  |  |  |  |  | |  | |  | |  | |
| DENV-1 infection (N=7) | 43 (7-83) | <0.01 | 91 (61-100) | 0.21 | 34 (0-84) | | <0.01 | | 20 (9-74) | | 0.08 | |
| DENV-3 infection (N=11) | 63 (0-98) | 0.02 | 85 (0-100) | 0.81 | 55 (23-100) | | <0.01 | | 32 (3-76) | | 0.31 | |
|  |  |  |  |  |  | |  | |  | |  | |
| (III) Non-acute DENV infection |  |  |  |  |  | |  | |  | |  | |
| Absence of neutralizing activity to all serotype (N=13) | 6 (0-22) | <0.01 | 5 (0-17) | <0.01 | 12 (0-29) | | <0.01 | | 11 (0-21) | | <0.01 | |
| Neutralizing activity to 1 DENV serotype (N=4^d^) | 52 (5-88) | 0.30 | 39 (9-100) | 0.32 | 17 (0-32) | | <0.01 | | 10 (3-12) | | <0.01 | |
| Neutralizing activity to 2 DENV serotype (N=7) | 75 (44-98) | 0.07 | 60 (0-100) | 0.19 | 57 (45-77) | | <0.01 | | 19 (6-38) | | <0.01 | |
| Neutralizing activity to ≥3 DENV serotype (N=31) | 92 (59-100) | -^c^ | 82 (30-100) | - | 92 (55-100) | | - | | 41 (3-100) | | - | |
|  |  |  |  |  |  |  | |  | |  | |  |

^a^ % or, mean plaque reduction (%) was calculated by the formula: [total (percentage of plaque reduction)/ number of patients], at 1:10 dilution, by using FcγR-negative BHK cells. Values in brackets indicate the lowest and highest percentage of plaque reduction (%).

^b^ P-value of plaque reduction (%) compared with those with neutralizing activity to ≥3 DENV serotype. Underline indicates P value of less than 0.05.

^c^ Indicates that calculation is not possible.

^d^ Serum sample #37 does not possess neutralizing activity to any DENV serotype, but exhibited infection-enhancement activity to DENV-3.
